# Clinical features, comorbidities, and therapeutic outcomes of Cushing syndrome: a retrospective multicenter study from Saudi Arabia

**DOI:** 10.3389/fendo.2026.1948006

**Published:** 2026-09-23

**Authors:** Mussa H. Almalki, Abdullah Alamri, Yara H. Musa, Rawan W. Kawsara, Aishah Ekhzaimy, Moeber Mahzari, Mohammed Z. Almutairi, Wadei Elhakimi, Abdulrahman Hummadi, Mohammed H. Alqambar, Saud Alzahrani, Ali S. Alzahrani

**Affiliations:** 1 Obesity, Endocrine, and Metabolism Center, King Fahad Medical City, Second Health Cluster, Riyadh, Saudi Arabia; 2 College of Medicine, Alfaisal University, Riyadh, Saudi Arabia; 3 Endocrinology and Diabetes Unit, Department of Medicine, College of Medicine and King Saud University Medical City, King Saud University, Riyadh, Saudi Arabia; 4 College of Medicine, King Saud bin Abdulaziz University for Health Sciences, Riyadh, Saudi Arabia; 5 King Abdullah International Medical Research Center, Riyadh, Saudi Arabia; 6 Department of Medicine, Ministry of National Guard Health Affairs, Riyadh, Saudi Arabia; 7 Department of Internal Medicine, Security Forces Hospital, Riyadh, Saudi Arabia; 8 Division of Endocrinology, Department of Internal Medicine, Almoosa Specialist Hospital, Al Mubarraz, Saudi Arabia; 9 Adult Endocrinology and Diabetes Center, Jazan Health Cluster, Ministry of Health, Jazan, Saudi Arabia; 10 Adult Endocrinology Unit, King Fahad Specialist Hospital, Dammam, Saudi Arabia; 11 Department of Medicine, King Faisal Specialist Hospital and Research Center, Jeddah, Saudi Arabia; 12 Molecular Endocrine Section, Research and Innovation, King Faisal Specialist Hospital and Research Center, Riyadh, Saudi Arabia; 13 Department of Medicine, King Faisal Specialist Hospital and Research Center, Riyadh, Saudi Arabia

**Keywords:** Cushing disease, Cushing syndrome, hypercortisolism, pituitary, Saudi Arabia, transsphenoidal surgery

## Abstract

**Purpose:**

Cushing syndrome (CS) is associated with significant morbidity and mortality, but data from the Middle East, including Saudi Arabia, are limited. This study aimed to characterize the demographics, clinical features, comorbidities, diagnostic findings, treatments, and outcomes in a Saudi Arabian multicenter tertiary-referral cohort.

**Methods:**

In this retrospective multicenter study, 146 patients with confirmed CS were included. Demographics, clinical features, laboratory and imaging results, treatments, and outcomes were collected and analyzed according to etiological subtype.

**Results:**

Among 146 patients (114 women, 32 men; mean age at last recorded follow-up or data abstraction, 42.2 ± 14.7 years), pituitary CS was the most common etiology (61.0%). The mean diagnostic delay was 7.5 years (median 5 years). Hypertension (62.3%) and dysglycemia (67.1%) were prevalent. Among the 88 patients who underwent bone mineral density assessment, 71.6% had abnormal bone health; this selected-subgroup estimate should not be generalized to the entire cohort. Hypokalemia was frequent in ectopic CS (84.6%). Treatment was etiology-specific: 85.4% of the patients with pituitary CS underwent transsphenoidal surgery (TSS), and 87.9% of patients with adrenal CS who underwent adrenalectomy had a unilateral procedure. Crude mortality was 5.1% (7/138 patients with known vital status), remission was documented in 66.9% (93/139 with recorded outcomes), and recurrence after a documented remission occurred in 12 patients.

**Conclusion:**

In this Saudi Arabian multicenter tertiary-referral cohort, CS predominantly affects women and is most frequently attributable to pituitary CS. Diagnostic delay remains prolonged despite advances in diagnosis and management. Descriptive treatment outcomes were broadly consistent with international experience; crude mortality was 5.1% among patients with known vital status.

## Introduction

Cushing syndrome (CS) is a rare and challenging endocrine disorder resulting from chronic exposure to high glucocorticoid levels, either from endogenous or exogenous glucocorticoid therapy (1). Endogenous CS is broadly classified into adrenocorticotropic hormone (ACTH)-dependent and ACTH-independent disease. ACTH-dependent CS includes Cushing disease (CD), caused by ACTH-secreting pituitary adenomas, and ectopic ACTH syndrome resulting from non-pituitary neuroendocrine tumors. ACTH-independent CS is most commonly caused by adrenal adenomas, adrenal carcinoma, or bilateral adrenal hyperplasia (2).

The reported annual incidence of endogenous CS ranges from approximately 1.2 to 2.4 cases per million population, with an estimated prevalence of 39–79 cases per million (3, 4). Despite its rarity, CS exerts a profound impact on health-related quality of life and carries a significantly elevated mortality burden, driven primarily by cardiovascular disease, thromboembolic events, infections, and immunosuppression (5, 6). The standardized mortality ratio (SMR) is 2 to 9 times higher than in the general population (2, 6), and even patients in remission may still face health risks (7–9).

A major challenge in CS is delayed diagnosis, with contemporary studies demonstrating that the interval from symptom onset to diagnosis often exceeds 2 years (10, 11). Early diagnosis and treatment are important because biochemical remission can reduce mortality and improve outcomes for related complications (12, 13). Surgery is the principal first-line treatment: transsphenoidal surgery (TSS) for CD, adrenalectomy for unilateral adrenal CS, and resection of an ectopic ACTH-secreting tumor when feasible. Remission rates after TSS for CD range from 53% to 80% in experienced centers (9, 14, 15). However, recurrence may occur during long-term follow-up and can require repeat surgery, radiotherapy, bilateral adrenalectomy, or medical therapy (1, 2, 16). Medical therapy is also important when surgery is contraindicated, delayed, unsuccessful, or followed by recurrence (17).

Large national and international registries, such as the European Registry on Cushing Syndrome (ERCUSYN), have provided detailed data from Western countries (18, 19). To date, no large, multicenter studies on CS have been published from the Arab world, especially Saudi Arabia. This study aims to fill that gap by providing, for the first time, a detailed description of the demographics, clinical features, comorbidities, biochemical and imaging findings, treatments, and outcomes of CS patients at several major academic hospitals across Saudi Arabia.

## Methods

### Study design and setting

This retrospective multicenter observational study was conducted at nine major tertiary care governmental and academic hospitals across Saudi Arabia. These institutions serve as the principal referral centers for complex endocrine disorders and provide specialist endocrine, radiology, endocrine surgery and neurosurgery services. All involved centers have been operating for more than 20 years. All endocrinologists involved in the care of patients with CS in Saudi Arabia were invited to submit their data. Data were collected directly from the medical records and assembled in a single data collection sheet. Diagnostic and follow-up practice was not centrally harmonized. Participating centers followed contemporary guideline-based practice, but assays, laboratory platforms, local reference ranges, imaging protocols, and follow-up schedules could differ by center and calendar period. No central laboratory reanalysis was performed. Recorded results were interpreted according to the assay-specific reference range and clinical assessment documented at the treating center; this heterogeneity was considered and addressed in the limitations.

### Patient population and diagnostic criteria

Patients of all age groups with confirmed endogenous CS were included. Diagnosis was based on the Endocrine Society Clinical Practice Guidelines (20). For this retrospective study, biochemical confirmation required at least two abnormal first-line tests documented in the medical record: failure to suppress serum cortisol to <1.8 µg/dL (50 nmol/L) on a 1-mg overnight dexamethasone suppression test (ODST), elevated 24-hour urinary free cortisol (preferably in at least two collections when available), and/or loss of diurnal cortisol rhythm with elevated late-night salivary cortisol. Etiologic subtype (ACTH-dependent vs. ACTH-independent) was determined using plasma ACTH, dynamic testing when required (high-dose DST or corticotropin-releasing hormone [CRH] stimulation), and anatomical imaging (pituitary magnetic resonance imaging [MRI] or adrenal computed tomography [CT]). Inferior petrosal sinus sampling (IPSS) was performed when indicated to confirm a pituitary source.

### Data collection

Data were extracted from electronic and hard-copy medical records using a standardized data extraction instrument. Variables collected included: demographics (age, sex, body mass index [BMI]); diagnostic delay (defined as the interval from symptom onset, as retrospectively documented in the medical record to confirmed diagnosis); biochemical parameters (baseline morning serum cortisol, UFC, ACTH, midnight serum and salivary cortisol); imaging findings such as tumour size; comorbidities; treatment modalities (first-line, second-line, and subsequent interventions); and clinical outcomes (remission, recurrence, persistent disease). Because symptom onset relied on patient recall and retrospective documentation, diagnostic-delay estimates were considered approximate.

Comorbidities were identified from documented diagnoses, relevant medication use, laboratory results, and imaging reports in the source records; they were not retrospectively re-evaluated using a uniform threshold across all centers or periods. Diabetes and prediabetes were based on the treating clinician’s recorded diagnosis, glucose-lowering treatment, and/or documented glycemic results interpreted according to contemporaneous local practice. Hypertension and dyslipidemia required a documented diagnosis and/or specific treatment. Infection included clinically diagnosed infections recorded during active CS (e.g., those requiring antimicrobial therapy and/or hospitalization). Osteopenia and osteoporosis were based on the interpreting DXA (dual-energy X-ray absorptiometry) report (generally T-scores < −1.0 to >−2.5 and ≤−2.5, respectively), whereas low bone mineral density for age was based on an age-appropriate Z-score interpretation (generally ≤−2.0). Fragility fracture was defined as a fracture occurring with low-energy trauma, such as a fall from standing height or less as documented in the record. As these definitions rely on retrospective documentation rather than prospective, protocol-driven ascertainment, some degree of misclassification is possible; this limitation is acknowledged in the discussion section.

### Outcome definitions

Treatment outcomes were classified using the 2021 international consensus guideline for CD where applicable (21) and etiology-appropriate biochemical criteria for other forms of CS. Early remission after pituitary surgery was defined as a morning serum cortisol concentration <138 nmol/L (5 µg/dL) within 7 days. Delayed remission was defined by subsequent normalization of morning cortisol, suppression after the 1-mg ODST (<50 nmol/L), and/or normalization of 24-hour UFC within 30 days after surgery. Following radiotherapy, remission required sustained biochemical normalization after withdrawal of cortisol-lowering medication whenever feasible. Persistent disease was defined as failure to achieve biochemical normalization after initial therapy. Recurrence was defined as biochemical hypercortisolism after a documented period of remission, with or without recurrent clinical features.

### Statistical analysis

All statistical analyses were conducted using SPSS version 26 (IBM Corp., Armonk, NY, USA). Descriptive statistics were summarized as frequencies and percentages for categorical variables and as mean ± standard deviation (SD) or median with interquartile range (IQR) for continuous variables, depending on the distribution. Comparisons of categorical variables across etiologic groups were performed using the Chi-square test or Fisher’s exact test, as appropriate. Continuous variables were compared using one-way analysis of variance (ANOVA) for normally distributed data and the Kruskal-Wallis H-test for non-normally distributed variables. *Post hoc* pairwise comparisons were performed when global tests were significant.

Analyses used available cases without imputation, and the denominator is reported when it differed from the full cohort. Because testing and follow-up were clinically selected, subgroup prevalence estimates—particularly DXA findings—were interpreted as estimates among assessed patients rather than the entire cohort. All comparisons were exploratory; two-sided p<0.05 was used without adjustment for multiple comparisons.

No recurrence-free survival analysis was retained because follow-up duration, a uniform time origin, and numbers at risk could not be reconstructed reliably from the aggregate dataset. Recurrence was therefore summarized descriptively among patients with a recorded last outcome.

## Results

### Patient demographics and diagnostic delay

A total of 146 patients with confirmed CS from nine major referral centers across Saudi Arabia were included. There were 114 women (78.1%) and 32 men (21.9%), a female-to-male ratio of 3.6:1. The overall mean age at the last recorded follow-up or data abstraction was 42.2 ± 14.7 years, whereas mean age at diagnosis was 35.0 ± 14.2. Age at diagnosis differed significantly by etiology (pituitary: 32.6 ± 11.2 years; adrenal: 36.4 ± 16.8 years, ectopic: 46.0 ± 18.3 years; F = 5.675, p=0.004). The mean diagnostic delay was 7.5 years, with median 5 years (IQR 2–10 years). Diagnostic delay was longest in pituitary CS (mean 8.9 years, median 6) compared with adrenal CS (mean 5.3 years, median 4) and ectopic CS (mean 5.6 years, median 4). In a descriptive analysis by diagnostic period, median delay decreased from 8 years before 2010 to 4 years after 2015 (p=0.03). Because the number and completeness of cases varied over time and diagnostic practices evolved, this temporal comparison should be interpreted as exploratory rather than as a causal effect of any specific service change. Baseline characteristics are summarized in **Table 1**.

**Table 1:** Baseline demographic and etiological characteristics of the patients.

Variable	Overall (n=146)	Pituitary (n=89)	Adrenal (n=44)	Ectopic (n=13)	Test statistic	P-value
Age in years, mean ± SD	42.2 ± 14.7	41.0 ± 12.6	41.8 ± 16.9	51.6 ± 17.3	F=3.072	0.049*
Age at diagnosis, years, mean ± SD	35.0± 14.2	32.6 ± 11.2	36.4 ± 16.8	46.0 ± 18.3	F=5.675	0.004*
Sex – Male, n (%)	32 (21.9%)	17 (19.1%)	8 (18.2%)	7 (53.8%)	χ^2^ = 8.515	0.014*
Sex – Female, n (%)	114 (78.1%)	72 (80.9%)	36 (81.8%)	6 (46.2%)		
BMI, kg/m², mean ± SD	32.1 ± 7.83	32.6 ± 7.63	32.0 ± 8.29	29.2 ± 7.63	F=1.002	0.370

BMI, body mass index. P-values by chi-square, Fisher’s exact, or one-way ANOVA test as appropriate. *Significant at p<0.05. “Age” denotes age at last available follow-up/data collection; “Age at diagnosis” denotes age at confirmed diagnosis of CS.

### Etiology

Pituitary CS was the most common etiology, accounting for 89 patients (61.0%), followed by adrenal CS in 44 patients (30.1%) and ectopic CS in 13 patients (8.9%). Most pituitary CS patients were female (80.9%), whereas males accounted for a higher proportion in ectopic CS (53.8%). Sex distribution differed significantly by etiology (χ² = 8.515, p = 0.014). The etiologic distribution by sex is presented in **Table 1**.

### Clinical manifestations

The predominant clinical features and their variation across etiological subgroups are summarized in **Figure 1** and **Supplementary Table S1**. Overall, weight gain (81.5%), rounded face (68.5%), central obesity (64.4%), proximal muscle weakness (55.5%), dorsocervical fat pad (54.8%), abdominal striae (48.6%), hirsutism (43.2%), and supraclavicular fullness (43.2%) were the most common clinical manifestations.

**Figure 1:**
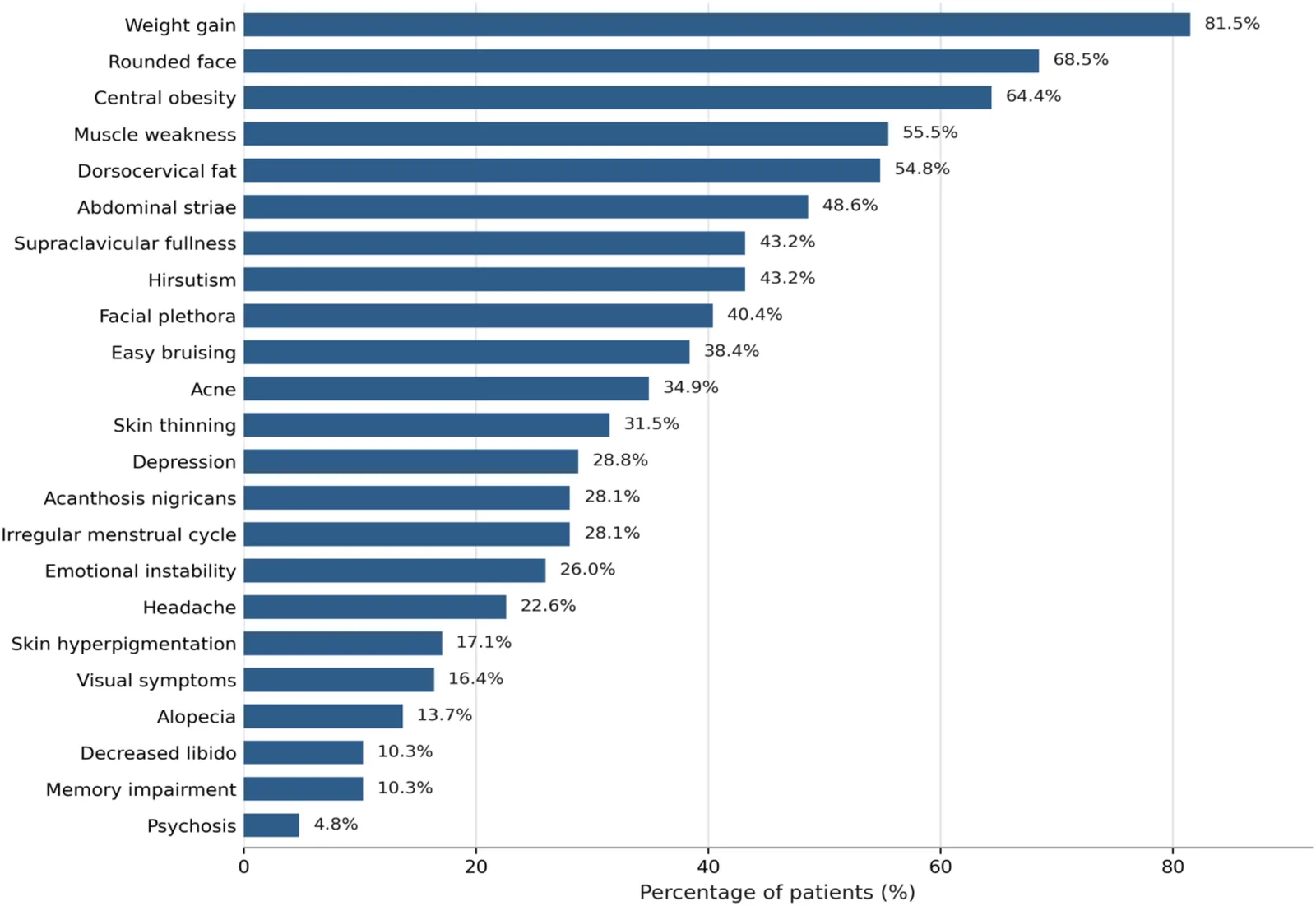
Prevalence of clinical manifestations at presentation (N = 146), ordered by frequency.

Several clinical features differed significantly between etiological subgroups, including weight gain, obesity, central obesity, dorsocervical fat pad, hirsutism, supraclavicular fullness, acanthosis nigricans, headache, visual symptoms, and psychosis (all *p* < 0.05). Headache and visual symptoms were most frequent in pituitary CS, consistent with tumor mass effects, whereas psychosis was more common in ectopic CS, likely reflecting more severe hypercortisolism.

### Comorbidities

Patients had a substantial burden of metabolic and systemic comorbidities (**Table 2**). Hypertension was present in 62.3%, and dyslipidemia in 39.7%. Among 143 patients with recorded glucose status, 75 (52.4%) had diabetes, 21 (14.7%) had prediabetes, and 96 (67.1%) had dysglycemia. Among the 88 patients (60.3% of the cohort) who underwent bone mineral density assessment, osteoporosis was identified in 31 (35.2%), low bone mineral density for age in 17 (19.3%), and osteopenia in 15 (17.0%). These categories accounted for 71.6% of assessed patients, but the estimate may be affected by selective testing and should not be interpreted as whole-cohort prevalence. Fragility fractures were documented in 22 patients (15.1%) in the entire cohort. Hypokalemia was documented in 30 patients (20.5%): 11/13 (84.6%) with ectopic CS, 10/44 (22.7%) with adrenal CS, and 9/89 (10.1%) with pituitary CS (p<0.001). Other listed comorbidities did not differ significantly among etiologic groups.

**Table 2:** Comorbidities in relation to etiology.

Variable	Overall (n=146)	Pituitary (n=89)	Adrenal (n=44)	Ectopic (n=13)	P-value
Hypertension, n (%)	91 (62.3)	50 (56.2)	31 (70.5)	10 (76.9)	0.154
Dyslipidemia, n (%)	58 (39.7)	34 (38.2)	20 (45.5)	4 (30.8)	0.570
Fatty liver, n (%)	24 (16.4)	14 (15.7)	8 (18.2)	2 (15.4)	0.941
Fragility fracture, n (%)	22 (15.1)	13 (14.6)	7 (15.9)	2 (15.4)	1.000
Infection, n (%)	18 (12.3)	10 (11.2)	4 (9.1)	4 (30.8)	0.128
DVT, n (%)	6 (4.1)	3 (3.4)	1 (2.3)	2 (15.4)	0.111
CAD, n (%)	5 (3.4)	2 (2.2)	2 (4.5)	1 (7.7)	0.355
Ischemic stroke, n (%)	4 (2.7)	4 (4.5)	0 (0.0)	0 (0.0)	0.519
PE, n (%)	4 (2.7)	1 (1.1)	2 (4.5)	1 (7.7)	0.116
Hypokalemia, n (%)	30 (20.5)	9 (10.1)	10 (22.7)	11 (84.6)	<0.001*
Prediabetes, n (%)	21/143 (14.7)	10/86 (11.6)	9/44 (20.5)	2 (15.4)	0.098
Diabetes, n (%)	75/143 (52.4)	47/86 (54.7)	18/44 (40.9)	10 (76.9)	
Osteopenia, n (%)	15/88 (17.0)	8/56 (14.3)	4/25 (16.0)	3/7 (42.9)	0.421
Osteoporosis, n (%)	31/88 (35.2)	19/56 (33.9)	10/25 (40.0)	2/7 (28.6)	
Low BMD for age, n (%)	17/88 (19.3)	11/56 (19.6)	4/25 (16.0)	2/7 (28.6)	

BMD, bone mineral density; DVT, deep vein thrombosis; CAD, coronary artery disease; PE, pulmonary embolism. *Significant at p<0.05.

### Biochemical and imaging investigations

Biochemical findings are summarized in **Supplementary Table S2**. As expected, plasma ACTH concentrations differed markedly by disease etiology (p<0.001), with the highest values observed in ectopic CS and the lowest in adrenal CS. Markers of hypercortisolism, including morning serum cortisol, 24-hour urinary free cortisol, and cortisol following both the 1-mg and 8-mg ODST also differed significantly between etiological subgroups (all *p* < 0.01), with the greatest cortisol excess observed in ectopic CS. Among additional laboratory parameters, DHEAS and triglyceride (TG) concentrations differed significantly across etiologies (both p<0.05), whereas renal function, HbA1c, fasting blood glucose (FBG), thyroid function tests, prolactin, Low-Density Lipoprotein cholesterol (LDL), and salivary cortisol were comparable between groups.

Radiological findings reflected the underlying disease subtype. Among patients undergoing pituitary MRI, microadenomas were identified in 48 patients (57.1%), macroadenomas in 36 (42.9%), and cavernous sinus invasion in 14/84 (16.7%). Among the 44 patients with adrenal CS who underwent adrenal CT, adrenal adenoma was identified in 30 patients (68.2%), adrenal carcinoma in 8 (18.2%), primary bilateral macronodular adrenal hyperplasia in 3 (6.8%), and primary pigmented nodular adrenocortical disease in 3 (6.8%).

### Treatment modalities and therapeutic outcomes

Treatment modalities varied significantly by etiology (**Table 3**). TSS was the primary treatment for pituitary CS (76/89, 85.4%), unilateral adrenalectomy accounted for 87.9% of adrenalectomies performed for adrenal CS. Medical therapy — particularly metyrapone (61.5%) and ketoconazole (38.5%) — was most commonly used in ectopic CS, whereas cabergoline was used predominantly in pituitary cases (40.4%). Osilodrostat, mitotane, pasireotide, and mifepristone were rarely used. Radiotherapy was used in 15.1% overall with no significant intergroup difference. Among 139 patients with recorded outcomes at last follow-up, remission was documented in 93/139 (66.9%): 54/82 (65.9%) with pituitary CS, 32/44 (72.7%) with adrenal CS, and 7/13 (53.8%) with ectopic CS. The overall distribution of remission, recurrence, and persistent disease did not differ significantly by etiology (p=0.080).

**Table 3:** Treatment modalities and therapeutic outcomes in relation to etiology at last follow-up.

Variable	Overall (n=146)	Pituitary (n=89)	Adrenal (n=44)	Ectopic (n=13)	P-value
TSS, n (%)	76 (52.1)	76 (85.4)	0 (0.0)	0 (0.0)	<0.001*
Unilateral adrenalectomy, n/N (%)^a^	30/51 (58.8)	1/12 (8.3)^b^	29/33 (87.9)^b^	0/6 (0.0)^b^	<0.001*
Bilateral adrenalectomy, n/N (%^a^	21/51 (41.2)	11/12 (91.7)^b^	4/33 (12.1)^b^	6/6 (100)^b^	—
Radiotherapy, n (%)	22 (15.1)	17 (19.1)	3 (6.8)	2 (15.4)	0.151
Ketoconazole, n (%)	27 (18.5)	20 (22.5)	2 (4.5)	5 (38.5)	0.003*
Metyrapone, n (%)	29 (19.9)	18 (20.2)	3 (6.8)	8 (61.5)	<0.001*
Osilodrostat, n (%)	2 (1.4)	2 (2.2)	0 (0.0)	0 (0.0)	1.000
Mitotane, n (%)	4 (2.7)	0 (0.0)	4 (9.1)	0 (0.0)	0.013*
Pasireotide, n (%)	1 (0.7)	0 (0.0)	0 (0.0)	1 (7.7)	0.088
Cabergoline, n (%)	38 (26.0)	36 (40.4)	1 (2.3)	1 (7.7)	<0.001*
Mifepristone, n (%)	1 (0.7)	1 (1.1)	0 (0.0)	0 (0.0)	1.000
Adrenal insufficiency, n (%)	50 (34.2)	22 (24.7)	23 (52.3)	5 (38.5)	0.007*
Outcome (n=139 with recorded outcome)^c^					0.080
Remission, n (%)	93 (66.9)	54 (65.9)	32 (72.7)	7 (53.8)	
Recurrence, n (%)	12 (8.6)	11 (13.4)	1 (2.3)	0 (0.0)	
Persistent active disease, n (%)	34 (24.5)	17 (20.7)	11 (25.0)	6 (46.2)	
Mortality (n=138 with known vital status)					0.051
Died, n (%)	7/138 (5.1)	2/83 (2.4)	3/44 (6.8)	2/11 (18.2)	

TSS transsphenoidal surgery; GH growth hormone. ^a^Denominator is patients who underwent adrenalectomy (n=51). ^b^Percentage of patients of that etiology who underwent adrenalectomy (not of the full etiological column). ^c^Seven patients (all pituitary CS) had no outcome recorded at last follow-up and are excluded from the outcome percentages. *Significant at p<0.05. Bilateral adrenalectomy in pituitary CS was used as definitive control for persistent or recurrent hypercortisolism when pituitary-directed treatment was unsuccessful or unsuitable.

Recurrence was documented in 11/82 (13.4%) of pituitary cases with recorded outcome, 1/44 (2.3%) adrenal cases, no recurrence was recorded in ectopic cases. Persistent active disease was most frequent in ectopic CS (46.2%). Follow-up duration could not be reconstructed consistently from the aggregate dataset and therefore time-dependent recurrence estimates were not retained. Adrenal insufficiency was documented in 24.7% of patients with pituitary CS, 52.3% with adrenal CS, and 38.5% with ectopic CS (p=0.007). In patients with pituitary CS, central hypothyroidism (31.0%), central hypogonadism (23.0%), and growth hormone deficiency (14.9%) were documented after pituitary surgery. Overall crude mortality was 5.1% (7/138 patients with known vital status) and was highest in ectopic CS (18.2%). An SMR was not reported because person-time and reference-population methods were insufficiently documented for reproducible estimation.

## Discussion

This multicenter study provides the first large, comprehensive description of endogenous CS from nine tertiary referral centers in Saudi Arabia. The findings characterize demographics, clinical presentation, comorbidity burden, treatment patterns, and outcomes across the major etiologic subtypes. The proportions of women (78.1%) and pituitary CS (61.0%) were broadly consistent with international cohorts, including ERCUSYN (18, 19). Ectopic CS was more evenly distributed by sex (53.8% male), consistent with reports that it is relatively more common in older men (7, 9). Because this was a referral-center cohort without population-based case ascertainment, incidence and prevalence could not be estimated reliably.

The mean diagnostic delay was 7.5 years (median, 5 years), longer than the approximately 2-year delay reported in a large meta-analysis and other international series (10, 11). Delay was greatest in pituitary CS, which often evolves through nonspecific metabolic, dermatologic, psychiatric, and musculoskeletal features. In Saudi Arabia, the high background prevalence of obesity, diabetes, and metabolic syndrome may further obscure early manifestations (22).

Diagnostic delay has decreased over time (median 8 years before 2010 vs 4 years after 2015, p=0.03), although a median of 4 years is still clinically unacceptable. This temporal improvement likely reflects several concurrent secular changes over the ~25-year study period, including greater awareness, improved access to endocrine testing and imaging, and expansion of specialist pituitary services. Because the pre-2010 and post-2015 subgroups are of unequal and modest size and the cut-points are somewhat arbitrary, this stratified analysis should be viewed as descriptive rather than a formal test of an era effect, and residual confounding by diagnostic period cannot be excluded; we have therefore avoided pooling data across the full study period without this caveat.

Comorbidity burden was high, but each estimate must be interpreted in light of record-based definitions and available-case denominators. Hypokalemia was a key discriminating feature of ectopic CS (84.6% vs. 22.7% in adrenal CS and 10.1% in pituitary CS; p<0.001), reflecting the severe cortisol excess typical of ectopic ACTH secretion. Severe cortisol excess can saturate renal 11β-hydroxysteroid dehydrogenase type 2, permitting cortisol-mediated mineralocorticoid receptor activation, renal potassium loss, sodium retention, and hypertension (2, 5, 10). The marked etiologic gradient is therefore biologically plausible and clinically useful: severe hypokalemia with rapidly progressive hypercortisolism should increase suspicion for ectopic ACTH secretion. Diabetes affected 52.4% of patients with recorded glucose status, and 67.1% had dysglycemia. The high rates of diabetes and prediabetes observed here likely reflect the combined effect of glucocorticoid-driven hepatic gluconeogenesis, peripheral (skeletal muscle and adipose) insulin resistance, and impaired pancreatic β-cell compensation, superimposed on the already high background prevalence of obesity and type 2 diabetes in the Saudi and wider Gulf population (22); this regional metabolic burden may be more relevant than international comparators when interpreting the elevated dysglycemia rate relative to European cohorts (18). Similarly, abnormal bone health is consistent with the direct suppressive effect of glucocorticoid excess on osteoblast differentiation and survival, upregulation of RANKL with consequent osteoclast activation, and glucocorticoid-associated hypogonadism, all of which favor bone resorption over formation (23, 24); regional data on baseline vitamin D status and bone health in the Saudi population, where vitamin D deficiency is common, may further compound this risk, although we did not have systematic vitamin D data to test this directly. Neuropsychiatric manifestations were also frequent (25, 26).

Crude mortality was 5.1% among patients with known vital status. This proportion cannot be directly compared with standardized mortality estimates from population-based cohorts because follow-up duration, age structure, person-time, and reference populations differ (9, 27, 28). A reproducible SMR could not be confirmed from the available aggregate data and was therefore not retained as a principal result.

Most deaths occurred in patients with adrenal carcinoma (2 cases) and malignant ectopic ACTH sources (2 cases), consistent with the known excess mortality from malignancy and uncontrolled hypercortisolism (8, 28). Beyond the malignancy-related deaths, the excess mortality in CS is generally attributed to cardiovascular disease accelerated by chronic hypercortisolism-driven hypertension, dyslipidemia, and insulin resistance; an increased risk of venous thromboembolism related to glucocorticoid-induced hypercoagulability; and susceptibility to severe infection secondary to glucocorticoid-mediated immunosuppression (5, 6). Although the specific cause of every death could not be adjudicated systematically, these established mechanisms warrant prospective evaluation in future regional registries.

Among patients with pituitary CS who had a documented outcome at the last follow-up, 65.9% were in remission. This figure represents the last recorded disease status following heterogeneous treatment pathways and should not be interpreted as, or directly compared with, remission rates after first TSS. The observed remission proportion at the last follow-up was within the broad range reported internationally (1, 9, 14, 15, 29). However, comparisons should be interpreted cautiously because of differences in patient characteristics, treatment pathways, follow-up duration, remission definitions, and analytical denominators. Importantly, our estimate reflects remission at the last recorded follow-up after any treatment rather than remission specifically following first TSS.

Surgical complications were not systematically captured in our dataset; therefore, the surgical safety profile of this cohort could not be directly assessed or compared with the complication rates reported in other surgical series (30).

## Strengths and limitations

Strengths include the multicenter inclusion of all major etiologic subtypes and broad geographic representation of Saudi tertiary practice. Limitations include the retrospective design, missing data, nonstandardized follow-up and biochemical assessment across centers, and potential referral bias. Small subgroup sizes—particularly for ectopic CS—limited statistical power and produced imprecise estimates. Diagnostic delay was calculated from the recorded date of symptom onset, which is inherently difficult to establish precisely from retrospective medical records and may be subject to recall or documentation bias; this should be considered when interpreting the absolute delay estimates. Comorbidity ascertainment relied on documentation of a clinician diagnosis or treatment in the medical record rather than uniform, protocol-driven testing of every patient for every comorbidity, which may have led to under- or over-ascertainment for some conditions. Surgical complications and first-operation remission were not captured with sufficient consistency for denominator-specific analysis. Finally, without population-based ascertainment, the cohort cannot provide valid national incidence or prevalence estimates.

## Conclusion

In this Saudi Arabian multicenter tertiary-referral cohort, pituitary CS was the most common cause of CS and diagnostic delay remained prolonged despite improvement over calendar time. Metabolic and skeletal morbidity was substantial, but incomplete and selectively obtained assessments require cautious interpretation. Hypokalemia was a hallmark of ectopic CS. Treatment outcomes were broadly comparable to international benchmarks, and crude mortality was 5.1% among patients with known vital status and could not be standardized. These findings support Gulf-region recommendations (31) and the development of multidisciplinary pituitary services and prospective national registries.

## Data Availability

The original contributions presented in the study are included in the article/**Supplementary Material**, further inquiries can be directed to the corresponding author/s.
