# Supplementary material for: Clinical features, comorbidities, and therapeutic outcomes of Cushing syndrome: a retrospective multicenter study from Saudi Arabia
Source: Front Endocrinol (Lausanne). 2026 Sep 23;17:1948006. doi: 10.3389/fendo.2026.1948006 (PMC13642127; doi:10.3389/fendo.2026.1948006)
Supplement: Supplementary file 1 [file Table1.docx]

# **Supplementary Material**

Supplementary Table S1 presents detailed clinical manifestations by etiology, and Supplementary Table S2 presents laboratory and imaging findings.

Table S1. Detailed clinical manifestations according to etiology

| Variable | Overall N (%) (n=146) | **Etiology** | | | Test statistic | P-value |
| --- | --- | --- | --- | --- | --- | --- |
|  |  | Pituitary N (%) (n=89) | Adrenal N (%) (n=44) | Ectopic N (%) (n=13) |  |  |
| Menstrual cycle ^‡^ |  |  |  |  |  |  |
| - Normal | 16 (11.0%) | 11 (12.4%) | 5 (11.4%) | 0 (0.0%) | Exact test | 0.837 |
| - Amenorrhea | 30 (20.5%) | 23 (25.8%) | 7 (15.9%) | 0 (0.0%) |  |  |
| - Irregular | 41 (28.1%) | 29 (32.6%) | 10 (22.7%) | 2 (15.4%) |  |  |
| Weight gain ^§^ | 119 (81.5%) | 78 (87.6%) | 36 (81.8%) | 5 (38.5%) | χ²=18.205 | <0.001 * |
| Rounded face ^§^ | 100 (68.5%) | 64 (71.9%) | 30 (68.2%) | 6 (46.2%) | χ²=3.490 | 0.175 |
| Obesity ^‡^ | 96 (65.8%) | 61 (68.5%) | 31 (70.5%) | 4 (30.8%) | Exact test | 0.026 * |
| Central obesity ^‡^ | 94 (64.4%) | 63 (70.8%) | 29 (65.9%) | 2 (15.4%) | Exact test | <0.001 * |
| Proximal muscle weakness ‡ | 81 (55.5%) | 50 (56.2%) | 21 (47.7%) | 10 (76.9%) | Exact test | 0.187 |
| Dorsocervical fat pad § | 80 (54.8%) | 58 (65.2%) | 17 (38.6%) | 5 (38.5%) | χ²=9.905 | 0.007 * |
| Abdominal striae § | 71 (48.6%) | 46 (51.7%) | 20 (45.5%) | 5 (38.5%) | χ²=1.048 | 0.592 |
| Hirsutism ^‡^ | 63 (43.2%) | 46 (51.7%) | 15 (34.1%) | 2 (15.4%) | Exact test | 0.017 * |
| Supraclavicular fullness ^‡^ | 63 (43.2%) | 47 (52.8%) | 14 (31.8%) | 2 (15.4%) | Exact test | 0.007 * |
| Facial plethora § | 59 (40.4%) | 39 (43.8%) | 15 (34.1%) | 5 (38.5%) | χ²=1.180 | 0.554 |
| Easy bruising ^‡^ | 56 (38.4%) | 34 (38.2%) | 18 (40.9%) | 4 (30.8%) | Exact test | 0.870 |
| Acne ^‡^ | 51 (34.9%) | 36 (40.4%) | 12 (27.3%) | 3 (23.1%) | Exact test | 0.235 |
| Skin thinning ^§^ | 46 (31.5%) | 27 (30.3%) | 14 (31.8%) | 5 (38.5%) | χ²=0.350 | 0.840 |
| Depression ^‡^ | 42 (28.8%) | 24 (27.0%) | 14 (31.8%) | 4 (30.8%) | Exact test | 0.817 |
| Acanthosis nigricans ^‡^ | 41 (28.1%) | 28 (31.5%) | 13 (29.5%) | 0 (0.0%) | Exact test | 0.043 * |
| Emotional instability ^‡^ | 38 (26.0%) | 24 (27.0%) | 10 (22.7%) | 4 (30.8%) | Exact test | 0.773 |
| Headache ^‡^ | 33 (22.6%) | 29 (32.6%) | 2 (4.5%) | 2 (15.4%) | Exact test | <0.001 * |
| Skin hyperpigmentation ^‡^ | 25 (17.1%) | 15 (16.9%) | 6 (13.6%) | 4 (30.8%) | Exact test | 0.361 |
| Visual symptoms ^‡^ | 24 (16.4%) | 21 (23.6%) | 1 (2.3%) | 2 (15.4%) | Exact test | 0.003 * |
| Alopecia ^‡^ | 20 (13.7%) | 15 (16.9%) | 5 (11.4%) | 0 (0.0%) | Exact test | 0.276 |
| Memory impairment ^§^ | 15 (10.3%) | 7 (7.9%) | 4 (9.1%) | 4 (30.8%) | χ²=6.551 | 0.038 * |
| Decreased libido ^‡^ | 15 (10.3%) | 8 (9.0%) | 5 (11.4%) | 2 (15.4%) | Exact test | 0.646 |
| Psychosis ^‡^ | 7 (4.8%) | 2 (2.2%) | 2 (4.5%) | 3 (23.1%) | Exact test | 0.017 * |

BMI = body mass index. Results are expressed as numbers and percentages. Percentages use the full cohort and etiologic-group denominators shown in the column headings, consistent with Figure 1. The menstrual-cycle comparison used available data from 87 women: 63 with pituitary CS, 22 with adrenal CS, and 2 with ectopic CS. § P-values were calculated using the chi-square test. ‡ P-values were calculated using Fisher’s exact test. *Significant at p<0.05.

Table S2. Laboratory and imaging characteristics according to etiology

| Variable | Overall (n=146) | Pituitary (n=89) | Adrenal (n=44) | Ectopic (n=13) | Test statistic | P-value |
| --- | --- | --- | --- | --- | --- | --- |
| Creatinine, µmol/L, mean±SD | 60.19±20.05 | 59.16±15.49 | 63.47±26.55 | 55.40±22.11 | F=0.986 | 0.376 |
| Hypokalemia, n (%) | 30 (20.5) | 9 (10.1) | 10 (22.7) | 11 (84.6) | Exact test | <0.001* |
| HbA1c, %, mean±SD | 6.91±1.77 | 6.87±1.76 | 6.67±1.70 | 7.98±1.88 | F=2.425 | 0.093 |
| Random glucose, mmol/L, mean±SD | 9.25±4.13 | 8.70±3.80 | 9.43±4.46 | 11.72±4.26 | F=2.372 | 0.099 |
| FBG, mmol/L, mean±SD | 6.85±2.54 | 6.72±2.65 | 6.75±2.02 | 8.18±3.36 | F=0.905 | 0.409 |
| LDL, mmol/L, mean±SD | 3.23±0.99 | 3.22±1.08 | 3.32±0.78 | 2.76±1.15 | F=0.668 | 0.515 |
| Morning serum cortisol, nmol/L, mean ± SD | 690.84±694.68 | 607.93±409.00 | 511.46±295.51 | 1804.27±1623.48 | F=21.293 | <0.001* |
| Evening serum cortisol, nmol/L, mean ± SD | 602.19±390.46 | 560.66±406.12 | 582.74±359.93 | 958.67±207.46 | F=1.421 | 0.257 |
| Cortisol, 1-mg ODST, nmol/L, mean±SD | 554.85±710.41 | 426.96±367.70 | 446.88±302.30 | 1715.82±1706.65 | F=22.232 | <0.001* |
| Cortisol, 8-mg ODST, nmol/L, mean±SD | 279.23±226.65 | 222.93±165.26 | 385.40±319.26 | 609.00±283.38 | F=5.860 | 0.007* |
| TG, mmol/L, median (IQR) | 1.51 (0.93–2.02) | 1.49 (0.87–1.98) | 1.48 (1.04–1.95) | 2.40 (1.98–2.92) | H=6.218 | 0.045* |
| FT4, pmol/L, median (IQR) | 12.95 (11.62–15.00) | 13.10 (11.79–15.05) | 12.80 (11.37–14.27) | 12.90 (9.82–13.28) | H=1.662 | 0.436 |
| TSH, mIU/L, median (IQR) | 1.15 (0.79–2.17) | 1.15 (0.82–1.96) | 1.31 (0.81–2.69) | 0.80 (0.70–1.30) | H=3.408 | 0.182 |
| DHEAS, µg/mL, median (IQR) | 5.16 (1.64–9.27) | 7.59 (4.35–13.62) | 0.99 (0.41–4.50) | 6.60 (5.90–6.70) | H=17.358 | <0.001* |
| Prolactin, ng/mL, median (IQR) | 14.30 (10.05–23.62) | 14.70 (10.20–23.10) | 14.00 (10.33–28.00) | 9.10 (6.70–13.95) | H=1.455 | 0.483 |
| ACTH, pg/mL, median (IQR) | 52.40 (9.25–112.85) | 68.15 (29.65–110.50) | 2.00 (1.30–4.85) | 206.50 (182.75–340.50) | H=70.953 | <0.001* |
| 24-hour urinary free cortisol, mg/day, median (IQR) | 480.00 (218.00–894.00) | 500.00 (255.30–1150.00) | 235.00 (114.62–644.50) | 715.00 (661.00–760.00) | H=13.369 | 0.001* |
| Salivary cortisol, nmol/L, median (IQR) | 14.76 (5.12–27.30) | 15.13 (6.31–25.60) | 6.98 (3.54–26.24) | 39.11 (39.11–39.11) | H=2.196 | 0.334 |
| MRI pituitary – microadenoma, n/N (%) ᵃ | — | 48/84 (57.1) | — | — | — | — |
| MRI pituitary – macroadenoma, n/N (%) ᵃ | — | 36/84 (42.9) | — | — | — | — |
| Cavernous sinus invasion, n/N (%) ᵃ | — | 14/84 (16.7) | — | — | — | — |
| CT adrenal – adenoma, n/N (%) ᵇ | — | — | 30/44 (68.2) | — | — | — |
| CT adrenal – carcinoma, n/N (%) ᵇ | — | — | 8/44 (18.2) | — | — | — |
| CT adrenal – PBMAH, n/N (%) ᵇ | — | — | 3/44 (6.8) | — | — | — |
| CT adrenal – PPNAD, n/N (%) ᵇ | — | — | 3/44 (6.8) | — | — | — |

Mean ± SD for normally distributed variables; median (IQR) for non-normally distributed variables. HbA1c = glycated hemoglobin; FBG = fasting blood glucose; LDL = low-density lipoprotein cholesterol; ODST = overnight dexamethasone suppression test; TG = triglycerides; FT4 = free thyroxine; TSH = thyroid-stimulating hormone; DHEAS = dehydroepiandrosterone sulfate; ACTH = adrenocorticotropic hormone; MRI = magnetic resonance imaging; CT = computed tomography; PBMAH = primary bilateral macronodular adrenal hyperplasia; PPNAD = primary pigmented nodular adrenocortical disease. ᵃPituitary imaging findings are reported for patients with pituitary CS who underwent pituitary MRI (n=84). ᵇAdrenal CT findings are reported for patients with adrenal CS (n=44). *Significant at p<0.05.
